# Risk of Malignant Transformation in Patients With Oral Lichen Planus or Lichenoid Lesions: A Retrospective Study of 323 Patients With a Minimum of 5‐Year Follow‐Up

**DOI:** 10.1111/jop.70139

**Published:** 2026-04-14

**Authors:** Mari Vehviläinen, Tuomas Selander, Tuula Salo, Maria Siponen

**Affiliations:** ^1^ Department of Oral and Maxillofacial Diseases, Faculty of Medicine University of Helsinki Helsinki Finland; ^2^ Science Service Center Kuopio University Hospital Kuopio Finland; ^3^ Research Unit of Population Health, Faculty of Medicine, Medical Research Center University of Oulu and Oulu University Hospital; Oulu Finland; ^4^ Department of Oral and Maxillofacial Diseases, Translational Immunology Program (TRIMM), iCAN Digital Precision Cancer Medicine Flagship University of Helsinki Helsinki Finland; ^5^ Faculty of Health Sciences, Institute of Dentistry University of Eastern Finland Kuopio Finland; ^6^ Odontology Education Unit and Department of Oral and Maxillofacial Diseases Kuopio University Hospital Kuopio Finland

**Keywords:** lichen planus, oral lichen planus, oral lichenoid lesions, oral squamous cell carcinoma

## Abstract

**Objective:**

To estimate the risk for oral squamous cell carcinoma (OSCC) among patients with oral lichen planus (OLP) or lichenoid lesions (OLL) and the possible risk factors.

**Material and Methods:**

Two hundred thirty‐nine OLP and 84 OLL patients diagnosed between years 1982 and 2017 were followed up to December 2023, diagnosis of an OSCC, or death. Potential demographic and clinical risk factors were compared, and a Cox proportional‐hazards model was used to investigate associations between time to OSCC onset and variables of interest.

**Results:**

Six OLP patients (2.5%; three females), in a mean follow‐up of 17.5 years, and six OLL patients (7.1%; three females), in a mean follow‐up of 13.8 years, developed OSCC. The annual MT rate was 0.14% for OLP and 0.5% for OLL. The mean time for OSCC development from OLP diagnosis was 11.4 years and from OLL diagnosis 12 years. Four OLP patients (67%; two females) developed OSCC in the mobile tongue, while in OLL patients OSCCs occurred at various sites. A potential association was observed between floor‐of‐mouth lesions in OLP and gingival or extraoral LP involvement in OLL and increased OSCC risk.

**Conclusion:**

This study confirms that both OLP and OLL patients are at a long‐term risk of OSCC, and the risk is higher for OLL patients. Site‐specific lesions in OLP and OLL were linked to increased OSCC risk, warranting confirmation in larger studies.

## 1 Introduction

Oral lichen planus (OLP) is a chronic inflammatory disease affecting 0.11%–3.2% of the worldwide population, with a higher frequency in middle‐aged females [1, 2]. OLP resembles oral lichenoid lesions (OLL) or reactions (OLR) both clinically and histopathologically. For OLL there are certain etiological factors, for example, reactions to drugs or dental materials (mainly amalgam) or conditions such as graft‐versus‐host disease (GVHD) [3, 4].

OLP is considered an oral potentially malignant disorder (OPMD) [5]. After the first report of possible transformation of OLP to oral squamous cell carcinoma (OSCC) in 1910, many studies have investigated the potential risk of malignant transformation (MT) of OLP [6, 7]. However, the MT potential of OLP remains controversial. In the earlier studies, the MT rate was high (even 5.6%) but some authors noted that the diagnosis of OLP was given incorrectly [8, 9]. Over the decades, several efforts have been made to overcome this issue. Most of the later studies have followed strict diagnostic criteria of OLP, with OLLs, erythroplakia, proliferative verrucous leukoplakia (PVL), and lichenoid dyplasia being excluded from the studies [7, 10]. According to the position paper of the AAOMP, the diagnosis of OLP is established if the criteria of both the clinical and histopathological features are met and if not, the lesions should be diagnosed as OLL [11]. For now, the reported MT rate of OLP is around 1% and for OLL the rate seems higher, 2.5% [12]. However, there is still significant controversy regarding the malignant potential of OLP and OLL [13].

Previously, the documented mean follow‐up time in studies investigating the MT risk of OLP and OLL has often been less than 5 years [12]. This affects the MT rate estimation, since OSCC occurs typically several years after the eruption of OLP/OLL [14, 15, 16]. Therefore, a long‐term follow‐up time is needed to establish the putative premalignant nature of OLP and OLL. Here, the patients were followed at least 5 years (60 months) to evaluate whether the MT risk of OLP and OLL increases over an extended follow‐up period. In addition, potential related risk factors were investigated.

## 2 Materials and Methods

We conducted a retrospective follow‐up study of 323 OLP or OLL patients who had been treated in Kuopio University Hospital (KUH) between 1982 and 2017. All cases who were given histopathological diagnoses of either OLP or OLL were analyzed. The diagnosis of OLP or OLL (including both oral lichenoid contact hypersensitivity reaction, lichenoid drug reaction and GVHD) was made by the first author (M.V.). by combining the histopathological and clinical features according to the classification by the American Academy of Oral and Maxillofacial Pathology (AAOMP) position paper [11].

The inclusion criteria were patients with: (1) confirmed diagnosis of OLP or OLL based on both clinical and histological parameters, according to the proposed diagnostic criteria of the AAOMP, and (2) an overall follow‐up of more than 5 years (60 months). The exclusion criteria were: (1) patients with other oral OPMD, dysplasia or previous oral malignancy, (2) patients who had moved out of the KUH area during the follow‐up time, and (3) patients with contact OLL caused by an identifiable cause, such as a hypersensitivity reaction to dental restorative materials or some other agent and if the lesions resolved after removal or replacement of the suspected causative factor. After these evaluations, the study group consisted of 239 OLP patients and 84 OLL patients.

At baseline, we collected demographic information from the patient charts: age, sex, and smoking status (smoker, non‐smoker or ceased smoking before OLP or OLL diagnosis). However, smoking status was divided later for the statistical analysis as either positive (smoking at the time of OLP/OLL diagnosis) or negative (not smoking at the time of OLP/OLL diagnosis). Alcohol consumption could not be reliably recorded. In addition, we recorded the medical history, including systemic diseases, regular medications, and allergies (both self‐reported and diagnosed by a dermatologist). A diagnosis of cutaneous LP (CLP) or genital LP (GLP) was given only if the lesions were diagnosed or suspected by a dermatologist (CLP) or gynecologist (GLP) or diagnosed as CLP or GLP by a physician (according to the patient) or suspected by a hospital dentist to be CLP. The localization of CLP was recorded.

Of the OLP and OLL, we listed clinical features of the lesions and sites of involvement. If the patient had several appointments, the same parameters had been recorded at each visit. OLP and OLL patients were divided into three groups based on lesion type: (1) patients with only white lesions (reticular, papular, plaque‐like), (2) patients with atrophic (red) lesions without erosive features, and with or without white lesions, and (3) patients with erosive lesions with or without any other clinical features (white or red). The sites of the lesions were defined as follows: buccal mucosa, gingiva, tongue, palatal mucosa, alveolar ridge mucosa, floor of mouth, and labial mucosa.

At the end of 2023, all OLP and OLL cases were reviewed, and MT to OSCC were documented, including the tumor location, TNM Classification, tumor grade, clinical type of OLP/OLL and possible previously diagnosed dysplasia on mucosa at same location before OSCC, interval from OLP/OLL diagnosis to OSCC, treatment modalities, and survival outcomes. The MT was registered when an OLP/OLL patient showed OSCC development at a previously verified OLP/OLL lesion and after a follow‐up duration of at least 6 months before the development of OSCC.

The start of the study period was defined as the day of the histopathological diagnosis of OLP/OLL. The cohort was followed up to December 28, 2023, diagnosis of an OSCC or death, whichever occurred first. The follow‐up started 6 months after OLP/OLL diagnosis aiming to exclude the possibility of a concomitant clinical presentation of OLP/OLL and OSCC [17]. Thus, the actual follow‐up began 6 months after the OLP diagnosis and continued for at least 5 years (60 months).

Periodic visits for all OLP/OLL patients were carried out either at KUH, health centers, or private clinics with a frequency based on the severity of mucosal disease and/or the need for treatment, as determined by a specialist, with at least annual follow‐up visits. In addition, patients were instructed to perform self‐monitoring and to contact the clinic immediately if suspicious lesions were detected. If OLP/OLL patients were suspected of a MT in primary care, the patient was referred to KUH for diagnosis and treatment.

The study protocol was approved by the Ethics Committee of the Northern Ostrobothnia Hospital District (7449/06.01.03.01/2013). KUH gave organizational consent for the study (215/2017).

Statistical analyses were performed with IBM SPSS Statistics 28 by the first author (M.V.). Descriptive statistics of OLP and OLL characteristics were described by frequencies and percentages or means and ranges. Basic characteristics between groups at baseline were compared by *t*‐tests and Pearson chi‐square tests. The possible risk factors in demographic and clinical features of studied patients were compared by ANOVA and Pearson chi‐square tests. A Cox proportional‐hazard model was performed for investigating the association between time‐to‐onset of cancer and the following factors of interest: gender, age, erosive lesions, smoking status, extraoral LP, and hypertension and hypercholesterolemia. *p* < 0.05 were considered statistically significant.

## 3 Results

### 3.1 Basic and Clinical Characteristics of the Study Patients

The demographic information, smoking status, follow‐up times, and clinical characteristics of the study patients are presented in Table 1.

**TABLE 1:** Basic and selected clinical characteristics of combined OLP/OLL, OLP, and OLL groups.

	All OLP/OLL patients (*n* = 323)	OLP patients (*n* = 239)	OLL patients (*n* = 84)	*p* OLP vs. OLL
Mean age (range)	56.5 (15–91)	55.14 (15–82)	60.33 (17–91)	0.003
	n (%)	n (%)	n (%)	
Gender				0.059
Female	219 (68)	169 (71)	50 (60)	
Male	104 (32)	70 (29)	34 (40)	
Smoking^a^				0.674
Non‐smokers	146 (68)	107 (66)	39 (72)	
Ex‐smokers	39 (18)	32 (20)	7 (13)	
Smokers	30 (14)	22 (14)	8 (15)	
Mean follow‐up time (median, range) in months	198 (168, 37–413)	210 (185, 54–413)	165 (150, 37–376)	
Patient group by lesion type^b^				0.184
White	90 (27.9)	61 (25.5)	29 (34.5)	
Atrophic	97 (30)	71 (29.7)	26 (31)	
Erosive	136 (42.1)	107 (44.8)	29 (34.5)	
Site of the lesion				
Buccal mucosa	286 (88.5)	222 (92.9)	64 (76.2)	< 0.001
Tongue	123 (38.1)	94 (39.4)	29 (34.5)	0.435
Floor of mouth	31 (9.6)	26 (10.9)	5 (6)	0.184
Gingiva	103 (31.9)	87 (36.4)	16 (19.0)	0.003
Palatinum	38 (11.8)	29 (12.1)	9 (10.7)	0.728
Alveolar ridge	37 (11.5)	27 (11.3)	10 (11.9)	0.880
Labial mucosa	32 (9.9)	28 (11.7)	4 (4.8)	0.067
Extraoral LP	73 (22.6)	61 (25.5)	12 (14.2)	0.034

*Note: p* values were calculated by ANOVA and chi‐square tests.

Abbreviations: LL, lichenoid lesions; LP, lichen planus; OLL, oral lichenoid lesions; OLP, oral lichen planus.

^a^ Information on smoking was missing from 33% (*n* = 78) of OLP patients and 36% (*n* = 30) of OLL patients.

^b^ The most severe clinical type was selected.

### 3.2 Squamous Cell Carcinoma

The prevalence of OSCC was higher in OLL (*n* = 6, 7.1%, three females) than in OLP group (*p* = 0.053). Six OLP (2.5%; three females) and six OLL patients developed OSCC during the follow‐up time (Table 2). Among the 12 patients who developed OSCC, nine were followed under specialist care at the University Hospital, two were subsequently transferred to primary care after initial specialist follow‐up, and one was managed exclusively in primary care following the diagnosis of OLP. The mean age of the patient at the time of OSCC diagnosis was 61.6 years (range 42.8–86.0) for OLP and 71.7 years (range 59.5–92.8) for OLL. In OLP patients, four out of six OSCCs developed in the mobile tongue, but in OLL patients the OSCCs developed in various sites. Patient number 12 with OLL and grave lesions on the alveolar ridge and palatinum refused biopsy and treatment when OSCC was suspected, and the cancer invaded the maxilla and nasal passage followed by lymph node metastasis. Therefore, the TNM classification and tumor grade are missing from that patient. The patient died of OSCC 9 months after the cancer diagnosis. Most of the patients (9/12) who developed OSCC were alive at the end of the follow‐up period (December 2023). Of note, one male patient with OLP and LP in the esophagus did not develop OSCC but was diagnosed with esophageal SCC (Grade 2, T3N2M0) 17 years after OLP diagnosis.

**TABLE 2:** Characteristics of OLP and OLL patients who developed OSCC.

Subject	OLP/OLL	Sex (M/F)	Age^a^ (years)	Smoking	Other comorbidities	OLP/OLL sites	Primary site of OSCC	Type of OLP/OLL before OSCC	Extraoral LP sites	Cancer grade	TNM^b^	Dysplasia^c^ before OSCC	Time from OLP/OLL diagnosis to OSCC (months)	Treatment modality
1	OLP	M	59	Ex‐smoker (15)^d^	Hypertension, hypercholesterolaemia	Buccal mucosa, gingiva	Buccal mucosa	Reticular, atrophic	No	1	cT2N0M0	No	142	Surgery
2	OLP	M	80	Ex‐smoker (40)^d^	Asthma, hypertension, diabetes	Buccal mucosa, gingiva, tongue, floor of mouth	Mobile tongue	Plaque, erosive	No	Microinvasive, grade missing	T1N0M0	No	244	Surgery
3	OLP	F	43	Ex‐smoker (10)^d^	No	Buccal mucosa, gingiva, tongue, alveolar ridge, labial mucosa	Mobile tongue	Reticular, atrophic, erosive	No	1	T1N0M0	No	102	Surgery, neck dissection
4	OLP	F	86	No	Hypertension, arthrosis, Lewy body disease, gout	Buccal mucosa, tongue, floor of mouth, alveolar ridge	Mobile tongue	Reticular, atrophic, plaque, erosive	Skin	3	cT1N0M0	No	54	Surgery
5	OLP	M	54	Ex‐smoker (10)^d^	Hypercholesterolaemia	Buccal mucosa, gingiva, tongue, palatinum, floor of mouth	Mobile tongue	Plaque	No	Microinvasive, grade missing	T1N0M0	No	99	Surgery
6	OLP	F	49	Smoker	No	Buccal mucosa	Buccal mucosa	Atrophic, erosive	Skin	1	T2N0M0	No	183	Surgery, neck dissection, radiation
7	OLL	M	60	No	No	Buccal mucosa, gingiva, tongue, palatinum	Gingiva/alveolar ridge, buccal sulcus	Reticular	Skin	2	T4N0M0	No	37	Surgery, neck dissection
8	OLL	F	81	No	Hypertension, hypothyroidism	Buccal mucosa, gingiva, tongue	Gingiva	Reticular, atrophic, erosive	Genital	2	T1N0M0	No	232	Surgery
9	OLL	M	93	No	Hypertension	Buccal mucosa, tongue, palatinum, alveolar ridge	Mobile tongue	Reticular, erosive	No	3	T1N0M0	No	311	Surgery
10	OLL	F	70	No	Hypertension, hypothyroidism, hypercholesterolaemia, coronary artery disease	Buccal mucosa, tongue, floor of mouth, alveolar ridge	Floor of mouth	Plaque, erosive	Skin	1	T1N0M0	Severe dysplasia	53	Surgery
11	OLL	M	65	Ex‐smoker (3)^d^	Hypercholesterolaemia, impaired fasting glucose	Buccal mucosa, gingiva	Buccal sulcus	Atrophic, plaque, erosive	Skin	2	T3N2BM0	Mild dysplasia	91	Surgery, neck dissection, radiation and chemotherapy
12	OLL	F	62	Smoker	No	Buccal mucosa, gingiva	Buccal sulcus, alveolar ridge, palatinum	Reticular	No	—	—	—	145	Palliative

Abbreviations: LP, lichen planus; OLL, oral lichenoid lesions; OLP, oral lichen planus; OSCC, oral squamous cell carcinoma.

^a^ Age at the time of OSCC.

^b^ Clinical staging was determined according to the TNM classification of the International Union Against Cancer (UICC).

^c^ Dysplasia diagnosed in the same location before OSCC diagnosis.

^d^ Years without smoking.

Two OLP patients with OSCC had also CLP, and three OLL patients with OSCC had CLP and one GLP (Table 2). In the OLP with OSCC group, four had quit smoking 10–40 years before OSCC diagnosis, one had never smoked, and one was a current smoker. In the OLL with OSCC group, four patients had never smoked, one was a current smoker, and one was an ex‐smoker (quit smoking 3 years before OSCC).

The annual MT rate was 0.14% for OLP, at a mean follow‐up of 17.5 years (210 months, SD 97.8) and 0.5% for OLL, at a mean follow‐up of 13.8 years (165 months, SD 78.8). The mean time for OSCC development from OLP diagnosis was 11.4 years (137.2 months, range 54–243.8) and from OLL diagnosis 12 years (144.8 months, range 37.1–311). None of the OLP patients and two of the OLL patients were diagnosed with epithelial dysplasia (mild, severe) prior to the OSCC diagnosis at the same location as the cancer developed.

The risk factors for OSCC are presented in Table 3. OLP lesions on the floor of the mouth seemed a potential risk factor for the development of OSCC (*p* = 0.002). In the OLL group, lesions on the gingiva appeared to increase the risk of OSCC (*p* = 0.002). Smoking was more common among OLL patients with than without OSCC; however, without statistical significance. In the OLL group, LP elsewhere in the body appeared as a possible risk factor for OSCC (*p* < 0.001). Hypertension and hypercholesterolemia were more frequently observed at the time of OLP or OLL diagnosis in patients who subsequently developed OSCC compared to those who did not, although the differences did not reach statistical significance.

**TABLE 3:** Selected patient characteristics compared between study groups with and without OSCC.

Variable	OLP without OSCC (*n* = 233)	OLP with OSCC (*n* = 6)	p‐value	OLL without OSCC (*n* = 78)	OLL with OSCC (*n* = 6)	*p*
Mean age (range)^a^	55.27 (15–82)	50 (33–81)	0.329	60.36 (17–91)	60 (49–68)	0.955
	n (%)	n (%)		n (%)	n (%)	
Gender			0.259			0.622
Male	67 (28.8)	3 (50)		31 (39.7)	3 (50)	
Female	166 (71.2)	3 (50)		47 (60.3)	3 (50)	
Hypothyroidism			0.347			0.256
Yes	30 (12.9)	0 (0)		12 (15.4)	2 (33.3)	
No	203 (87.1)	6 (100)		66 (84.6)	4 (66.7)	
Hypertension			0.214			0.228
Yes	63 (27)	3 (50)		21 (26.9)	3 (50)	
No	170 (73)	3 (50)		57 (73.1)	3 (50)	
Hypercholesterolaemia			0.146			0.256
Yes	30 (12.9)	2 (33.3)		12 (15.4)	2 (33.3)	
No	203 (87.1)	4 (66.7)		66 (84.6)	4 (66.7)	
Extraoral LP	59 (25.3)	2 (33.3)	0.657	8 (10.3)	4 (66.7)	< 0.001
Allergy			0.814			0.515
Yes	87 (37.3)	2 (33.3)		17 (21.8)	2 (33.3)	
No	146 (62.7)	4 (66.7)		61 (78.1)	4 (66.7)	
Smoking status^b^			0.822			0.245
Positive	21 (13.5)	1 (16.7)		7 (14.6)	2 (33.3)	
Negative	135 (86.5)	5 (83.3)		41 (85.4)	4 (66.7)	
Patient group by lesion type			0.323			0.228
White	61 (26.2)	0 (0)		28 (35.8)	1 (16.7)	
Atrophic	69 (29.6)	2 (33.3)		25 (32.1)	1 (16.7)	
Erosive	103 (44.2)	4 (66.7)		25 (32.1)	4 (66.7)	
Site of OLP/OLL involvement^c^						
Buccal mucosa	216 (92.7)	6 (100)	0.492	58 (74.4)	6 (100)	0.155
Tongue	90 (38.6)	4 (66.7)	0.165	25 (32.1)	4 (66.7)	0.086
Floor of mouth	23 (9.9)	3 (50)	0.002	4 (5.1)	1 (16.7)	0.250
Gingiva	83 (35.6)	4 (66.7)	0.119	12 (15.4)	4 (66.7)	0.002
Palatinum	28 (12)	1 (16.7)	0.731	7 (9)	2 (33.3)	0.063
Alveolar ridge	25 (10.7)	2 (33.3)	0.084	8 (10.3)	2 (33.3)	0.093
Labial mucosa	27 (11.6)	1 (16.7)	0.703	4 (5.1)	0 (0)	0.570

*Note: p* values were calculated by ANOVA and chi‐square tests.

Abbreviations: LP, lichen planus; OLL, oral lichenoid lesions; OLP, oral lichen planus; OSCC, oral squamous cell carcinoma.

^a^ Age at OLP/OLL diagnosis.

^b^ Information on smoking was missing from 33% (*n* = 78) of OLP patients and 36% (*n* = 30) of OLL patients. Smoking status was positive if a person was a smoker at the time of OLP/OLL diagnosis.

^c^ All OLP/OLL lesion sites were registered.

Extraoral LP among OLL patients indicated an increased risk of developing an OSCC (*p* = 0.017) (Table 4). In addition, erosive lesions, hypertension, and hypercholesterolemia among OLP and OLL patients associated with a higher risk of developing OSCC; however, the findings were not significant. Smoking among the OLL patients associated with a higher risk of developing OSCC without statistical significance.

**TABLE 4:** Hazard ratios (HRs) with 95% confidence intervals (CIs) and *p* values for selected demographic and clinical characteristics associated with OSCC onset.

Variable		OLP		OLL	
		HR (95% CI)	*p*	HR (95% CI)	*p*
Gender	F vs. M	0.41 (0.07–2.29)	0.243	1.31 (0.12–13.85)	0.825
Age^a^		0.95 (0.87–1.04)	0.714	1.01 (0.9–1.13)	0.899
Erosive lesions	Yes vs. no	2.53 (0.37–17.22)	0.343	7.6 (0.18–328.17)	0.219
Smoking status	Yes vs. no	1.98 (0.17–22.97)	0.586	19 (0.42–868.2)	0.131
Extraoral LP	Yes vs. no	1.82 (0.28–11.79)	0.528	25.85 (1.8–370.38)	0.017
Hypertension	Yes vs. no	5.96 (0.61–57.94)	0.124	3.05 (0.3–31.43)	0.348
Hypercholesterolaemia	Yes vs. no	2.27 (0.32–16.16)	0.413	6.67 (0.15–294.66)	0.327

Abbreviations: LP, lichen planus; OLL, oral lichenoid lesions; OLP, oral lichen planus.

^a^ Age at OLP/OLL diagnosis.

## 4 Discussion

The MT rate of OLP is a controversial issue and there are only a few studies reporting the risk of OSCC of OLL [12, 13, 18]. We used strict diagnostic criteria [11] to differentiate OLP from OLL patients and included only patients with at least a 5‐year follow‐up time. In most of the previous studies on MT in OLP and OLL, the follow‐up times have been shorter [5, 18, 19]. Applying these criteria, the MT rate of OLP was 2.5%, and for OLL it was even higher, 7.1%, even though the cases with dysplasia at the time of OLP or OLL diagnosis were excluded. However, the difference in MT rate between the groups was statistically non‐significant. This finding is in line with Ramos‐Garcia et al. [18] who found that OSCC rate ranged between 0.44% and 2.28% for OLP and between 1.88% and 3.80% for OLL, both being lower than in our study. However, a study by Aguirre‐Urizar et al. [19] reported MT rates of 1.7% for OLP and 5.9% for OLL, which are comparable to our findings, even though they had a substantially shorter mean follow‐up time of 72 months (6 years) vs. our 210 months (17.5 years) for OLP and 165 months (13.8 years) for OLL. Moreover, we observed that MT occurred in a mean time of 11.4 years (137 months) for OLP and 12.0 years (145 months) for OLL after initial OLP/OLL diagnosis. In comparison, a meta‐analysis [12] reported mean times from OLP diagnosis to MT ranging between 9.7 and 168 months. Based on our data we suggest that OLP and OLL patients should be followed up at least annually lifelong since the OSCC transformation may occur decades after the initial OLP or OLL diagnosis.

For OLP patients, we found an annual MT rate of 0.14% which is in line with the other studies (> 0.1%–0.69%) [12]. Although the MT rate for OLL (7.1%) in the present study was high, the annual MT rate for OLL was only 0.5%. Van der Meij et al. [20] reported a higher annual MT rate for OLL (0.71%). We believe it is more useful to estimate the annual MT rate instead of the MT rate in general.

The association between tobacco smoking and MT of OLP is unclear. Since smoking is carcinogenic, the main question is whether it can independently cause OSCC or does it interact with OLP to increase its malignant potential [12]. According to the modified WHO (2003) criteria, smoking should be correctly documented to differentiate true MT from cases of conventional OSCC that occur in OLP patients [10]. In meta‐analyses, smoking and alcohol consumption are associated with a higher risk of oral cancer among OLP patients [5, 18]. In the present study, smoking did not emerge as a significant risk factor for MT. However, approximately one‐third of cases lacked complete information on smoking status, which may have influenced the findings. Among OSCC cases in the OLP group, only one patient was a current smoker, while four had a history of smoking but had quit 10–40 years prior to OSCC diagnosis and even before the OLP diagnosis. In the OLL group, one patient was a current smoker, one had quit smoking three years prior to OSCC diagnosis, and the remaining patients had never smoked. Although smoking is an established independent risk factor for head and neck cancer, the risk decreases substantially after cessation, by approximately 50% for every 10 years of cessation, and approaches that of never‐smokers after long‐term cessation (> 20 years) [21]. Given the low proportion of current smokers and limited exposure among patients who developed MT in our cohort, smoking does not appear to have significantly contributed to MT in this data.

The most common location for OSCC among OLP patients was the mobile tongue, where the risk of OSCC development in general is higher than in any other location in the oral cavity [5, 18]. The locations of OSCCs were comparable to a recent study with a similar amount of OLP and OLL cases: most of the OSCCs were in the tongue in OLP patients and were seen in various locations in OLL patients [19]. Interestingly, in our study, a possible risk factor for OSCC in OLP patients was lesion location on the floor of the mouth instead of the tongue. Among OLL patients, lesions located in the gingiva were a potential risk factor for MT.

The atrophic‐erosive lesions of OLP/OLL have been shown to be a significant risk factor for MT [5, 18], and a meta‐analysis of reticular white lesions suggested no risk of MT [5]. In the present study, none of the clinical types of OLP or OLL were a significant risk factor for OSCC; however, erosive lesions were more common among patients who developed OSCC than those who didn't. One OLP patient had plaque‐like lesions and two OLL patients had reticular lesions on the OSCC location before OSCC diagnosis. Therefore, our results suggest that white OLP/OLL lesions may also develop into OSCC. However, it is noteworthy that none of the OLP patients, and only one OLL patient, who presented exclusively with white lesions, developed OSCC.

Most of the OLL patients with OSCC had LP elsewhere in the body, and it was a potential risk factor for OSCC. However, the mechanism behind this association remains unclear. It is possible that some of these patients might have OLP instead of OLL but for some reason either the clinical or histopathological features were atypical. Since lichenoid drug reactions can also present on the skin and genitals, it is possible that certain drugs could have caused these lesions, mimicking LP. None of our OLL patients had confirmed drug reactions, however, and the possible long latency time makes the diagnosis difficult [22]. Three out of six OLL patients with OSCC had medications which have been associated with lichenoid drug reactions [22, 23]. Usually, however, the lichenoid areas on the skin are larger than in LP, but the clinical features can resemble LP or be atypical [24]. Whether a possible drug reaction could have increased the malignancy risk is unknown. However, it might be beneficial to monitor OLL patients with extraoral LP/LL more carefully.

The strengths of this study were that all patient data were gathered and the diagnosis of OLP/OLL was rendered by a clinical specialist with strict criteria combining clinical and histopathological presentations. In addition, all patients had been diagnosed and treated in the same clinic during a 35‐year period. The follow‐up time in this study was long, at least 5 years, and comprehensive information on the clinical features of OLP/OLL patients was gathered from the medical files. The limitations of this study include the relatively small sample size and retrospective design, which may have influenced the accuracy of the available medical information. Furthermore, as all patients were referred to a university hospital for OLP or OLL diagnosis, a selection bias toward more severe cases is likely, which may in part account for the comparatively high MT rates observed. Patients with OLP and OLL managed in hospital‐based specialist care may represent a subgroup with more severe or extensive disease compared to those followed in primary care. High‐risk features, such as erosive or atrophic lesions, persistent symptoms and lesions, and wide oral involvement, might increase the risk of MT to OSCC and explain the higher incidence of OSCC in hospital‐based cohorts.

In conclusion, both OLP and OLL patients had an increased risk for OSCC, and the risk was higher, although not significantly, for OLL patients. In this limited material, the risk factors for OSCC among OLP patients appeared to be lesions on the floor of the mouth, and among OLL patients, lesions in the gingiva and extraoral LP. Further studies with a larger sample size are needed to examine the possible association between OLL and OSCC and extraoral LP lesions.

## Author Contributions

M.V., M.S., and Tuula Salo designed the study. M.V. gathered and analyzed the data. M.V., M.S., Tuula Salo, and Tuomas Selander performed interpretation of the results. M.V. wrote the original draft. M.S., Tuula Salo, and Tuomas Selander provided critical advice, manuscript editing, and other support to the study. All authors have approved the final version.

## Funding

The study was supported by a grant from The Finnish Medical Foundation.

## Ethics Statement

The study protocol was approved by the Ethics Committee of the Northern Ostrobothnian Hospital District (7449/06.01.03.01/2013).

## Conflicts of Interest

The authors declare no conflicts of interest.

## Data Availability

The data that support the findings of this study are available on request from the corresponding author. The data are not publicly available due to privacy or ethical restrictions.
